# Do wild-caught fly larvae cooperatively forage?

**DOI:** 10.1007/s00359-024-01724-3

**Published:** 2024-11-26

**Authors:** Rives Kuhar, Madeline Williamson, Peyton Yee, Guzel Naik, Sean Michael Cursain, Barry Condron

**Affiliations:** Department of Biology, University of Virginia, Charlottesville, VA 22901 USA

**Keywords:** Drosophila, Foraging, Cooperation, Social behavior, Group membership, Fitness, Wild type behavior

## Abstract

Animals often form organized cooperative foraging groups, where individual members must adhere to specific rules to maintain cohesiveness. These groups face the challenge of managing potential intruders, who may or may not assist in foraging. In semi-liquid food environments, *Drosophila* larvae learn to synchronize their movements into clusters, which are thought to make feeding more efficient. Individuals who do not synchronize with the group are excluded from the cluster. Whether clustering behavior occurs in wild-caught larvae, and if so, the extent of their selectivity in group membership, remains unknown. Here, we show that clustering occurs across a number of fly species, and the capacity to join different clusters varies both between and within species. We collected and observed a larval cluster from rotting fruit in the field, yielding seven fly species. Subsequent tests for clustering on five lines from this collection and 20 other inbred wild-caught lines revealed that all species, except *D. suzukii*, exhibit clustering behavior. Each line demonstrates varying capacities to become members of different clusters. This study also indicates that there is high genetic variance in how individual lines cluster with each other that is not explained by cross species features. Additionally, combinations of wild species with lab benchmark strains give varied outcomes in resultant adult fitness. The ability to co-cluster varies between and within species boundaries. However, fly lines that cluster with another tend to impart fitness both to themselves and their host. Our findings demonstrate that multiple species of fly larvae can co-cluster. This behavior tends to confer mutual benefits to cluster members, suggesting significant ecological implications in *Drosophila* communities.

**Supplementary Information:**

The online version contains supplementary material available at 10.1007/s00359-024-01724-3.

## Introduction

Cooperation makes sense when the value of a group exceeds that as an individual (Lewontin 1961; Maynard Smith 1982). The ideas of cooperative aggregations of animals has been a long-studied subject (Allee 1927). A critical early postulate of cooperative interactions is to determine whether they are ‘accidental’ or ‘essential’ (Deegener 1918). In ‘accidental’ cooperation the benefits of the group are small and more a consequence of crowding and co-existence. ‘Essential’ cooperation is the equivalent of a mutually supportive interaction and the fitness benefits are spread amongst the members (Deegener 1918). In addition, long term cooperative groups will very likely alter their interactions and this becomes a confound in understanding any model (Segraves et al. 2005; Rebar et al. 2020). It is essential to develop experimental models in which all of the individual behaviors and the resulting fitness are measurable.

Fly larvae have long provided an excellent model to study cooperation. On a rotting carcass, much attention has been focused on how larvae will secrete digestive enzymes and exodigest the substrate. In normal crowded conditions, exodigestion becomes communal and cooperative (Charabidze et al. 2021). Such cooperative exodigestion often includes multiple species and can involve structural organization of how individual larvae behave. However, in such a system, it becomes difficult to understand large groups of individuals with varied behavior. Small isolated groups of larvae can also establish (Beaver 1977). The larva of fruit fly *D.melanogaster* engages in social exodigestion (Gregg et al. 1990) and can form distinct coordinated foraging groups called clusters (Dombrovski et al. 2017, 2019, 2020; Khodaei 2019; Williamson et al. 2021). These clusters provide growth benefits (Dombrovski et al. 2020) and may also be protective for predation (Carton and David 1985). With a complete synaptic connectome (Winding et al. 2023) and experimental access to most of the neurons, the fly model heralds a great potential to understand complex social behavior.

The fruit fly *Drosophila melanogaster* has a rich history both in the lab and with studies in the field (Behrman et al. 2015; Gleason Id et al. 2019) and is largely thought to follow human migration (Keller 2007). While fruit flies are traditionally thought to eat rotting fruit (Becher et al. 2012), they are also able to survive on their own carcasses (Gregg et al. 1990), various meats (Yang 2018) and even human corpses and feces (Markow 2015). Like many non-eusocial insects, flies exhibit rich social behavior (Ferreira and Moita 2019; Couzin-Fuchs and Ayali 2021). Many studies have been carried out in the wild and brought back to the lab to look at inter species competition, especially between *D.melanogaster* and *D.simulans* (Moore 1952; Lewontin and Matsuo 1963; Sokolowski and Hansell 1983) which are thought to broadly co-exist. In addition, more recent interest has focused on potential interactions between *D.melanogaster* and the recently emerged pest flies *D.suzukii* and *Zaprionus indianus* (Lasa and Tadeo 2015; Shaw et al. 2018). While studies have concluded that there is intra- and inter-specific larval competition on food sources, not much is known about whether there could be cooperation. Understanding cooperation is essential towards a better handling of both endogenous and invasive fly species.

On semi-liquid food, fly larvae form cooperative clusters (Dombrovski et al. 2017; Khodaei 2019; Shoot et al. 2024). These clusters are characterized by larvae all orienting in a particular direction and synchronizing their movements so as to maintain even and continuous access to air via their posterior breathing spiracles while digging down into the substrate. At the same time, the food in front of these animals is communally digested and mixed. This behavioral phenotype is learned and involves a social critical period as well as plasticity in a visual circuit (Dombrovski et al. 2019) and imparts fitness to the individuals in adulthood (Dombrovski et al. 2020). Two important considerations when studying these behavioral structures is whether they are lab artifacts and how such groups could exist in the wild given the likelihood of many different species of larvae on the same substrate. Lab studies indicate that larvae grown under different temperatures than their peers (Williamson et al. 2021) or in social isolation are excluded from clusters resulting in lower fitness at high densities (Dombrovski et al. 2020). A simple hypothesis is that clusters in the wild would consist of a pure similarly developed set of single species larvae. Most lab strains cluster (Dombrovski et al. 2017, 2019, 2020; Williamson et al. 2021; Liao et al. 2024) but it remains unclear if wild fly larvae cluster. Furthermore, if wild caught larvae do cluster, how do the parameters of clustering vary? Since measuring clustering parameters is currently only possible in the lab (Liao et al. 2024) a cluster was found in the field and lines formed from the individual constituent larvae hatched to adults. These were used to examine how larvae in the wild might cluster. The purpose of this study was to ‘capture’ a single wild cluster and experimentally parse out the different members. A cluster was observed in the wild in this study, collected and found to contain many species of flies. Each of the members were inbred to produce a stable line, and controlled clustering experiments were carried out. The conclusions are that clusters can be multispecies, with mutual fitness exchange, but that variation of cooperative behavior also exists.

## Methods

### Processed food vials

All larvae were maintained on pre-processed food vials as described (Dombrovski et al. 2017). Pre-processed vials are those that have been about half liquified by CantonS (CS) larvae. This simulates a mature larval-populated piece of fruit. About 50 adult CS flies are placed in a Caltech-recipe food vial for 24 h during which about 100 eggs are laid. The adults are removed and the eggs allowed to hatch and pupate. At this stage the liquified front is about 50% down the vial. These are frozen, which kills all pupae and remaining larvae both of which remain in the vial.

### Collection

The outdoor collection experiments were carried out in a suburban shaded location in Central Virginia (Lat 38.006700, Long −78.534630) in August 2019. The temperature and humidity during this time were recorded and published elsewhere (Williamson et al. 2021). The tomato used for collection were Brandywine Heirloom cultivar. One single cluster was observed on a liquified tomato and collected. The cluster and about 1cm of surrounding medium were removed and placed in a vial and grown at 24 ˚C. To generate lines, single species male/female adult pairs were identified and paired after two generations of mixed growth in vials following field collection. Adults were used as the larvae observed in this study were morphologically indistinguishable. From each of these single paired matings, single male/female adult pairs were again chosen and mated. This was continued for 10 generations to yield inbred lines (Dr. Alan Bergland, U. of Virginia, pers comm).

### 2D clustering method

The 2D clustering assays were performed as described (Dombrovski et al. 2017). Liquified preprocessed food was placed between two glass slides and playdough spacers and 40 mid third instar larvae were placed on top. These were videorecorded with a iPhone 4s for 24 h. Clusters were defined as described (Dombrovski et al. 2017) and consist of 4 or more aligned larvae with at least a half a body length inserted into the food, and having made at least one synchronous vertical movement. The number of larvae in clusters at 4,5 and 6 h was averaged to determine the proportion of clustering. For transplantation, mid-third instar clustering animals were placed in blue food coloring for 30’, washed and then transferred to a 2D cluster. Cluster membership time was video recorded and measured from cluster entry to cluster exit as described (Dombrovski et al. 2017). For synchronization measures, a Nikon D3100 CMOS camera, with 50mm lens and fitted with a Raynox Macroscopic 4 × lens was used and videos (1920 × 1080 pixels) were recorded at 24Hz. Synchronization was measured between pairs of larvae in a cluster as described (Dombrovski et al. 2017).

### Wings

Newly hatched female adult flies were frozen and wings were removed, mounted and photographed as described (Dombrovski et al. 2020). All flies had a similar proximal to L3 set of markers to measure the major axis length except *Megaselia scalaris*. There was no clear L3 marker on these wings and so the longest distance from the proximal point to the tip of the wing was used.

### Stocks used

**Table:** 

Designation	Description	Species	Notes
* CS	CantonS	*D.melanogaster*	Ed Lewis, Caltech
* P	White-eyed host	*D.melanogaster*	Bloomington #24,055
* M1	12BME10, Maine	*D.melanogaster*	Alan Bergland, UVa
M8	12BME10.225, Maine	*D.melanogaster*	Alan Bergland, UVa
M9	12BME10.159, Maine	*D.melanogaster*	Alan Bergland, UVa
P4	LNPA-45, Pennsylvania	*D.melanogaster*	Alan Bergland, UVa
P7	LNPA-32, Pennsylvania	*D.melanogaster*	Alan Bergland, UVa
C1	CMH09.164, Virginia	*D.melanogaster*	Alan Bergland, UVa
C4	CMH09.095, Virginia	*D.melanogaster*	Alan Bergland, UVa
C5	CMH09.130, Virginia	*D.melanogaster*	Alan Bergland, UVa
* DmR	DMelRF, Virginia	*D.melanogaster*	This study
* C8	DMelCM8, Virginia	*D.melanogaster*	Alan Bergland, UVa
F1	Florida4,27	*D.melanogaster*	Alan Bergland, UVa
F2	Florida4,12	*D.melanogaster*	Alan Bergland, UVa
G1	Georgia13,29	*D.melanogaster*	Alan Bergland, UVa
G2	Georgia13,24	*D.melanogaster*	Alan Bergland, UVa
Ms1	Mississippi 24,3	*D.melanogaster*	Alan Bergland, UVa
Ms2	Mississippi 24,9	*D.melanogaster*	Alan Bergland, UVa
Ms3	Mississippi 24,2	*D.melanogaster*	Alan Bergland, UVa
* simR	DsimRF, Virginia	*D.simulans*	This study
* sim2	DsimCM85, Virginia	*D.simulans*	Alan Bergland, UVa
sim3	DsimCM74, Virginia	*D.simulans*	Alan Bergland, UVa
* meg	DSFRF, Virginia	*Megaselia scalaris*	This study
* zap	DDzapRF, Virginia	*Zaprionus indianus*	This study
* suz	DDsuzRF, Virginia	*D.suzukii*	This study

* Denotes those chosen for further study.

## Results

**Fig. 1:**
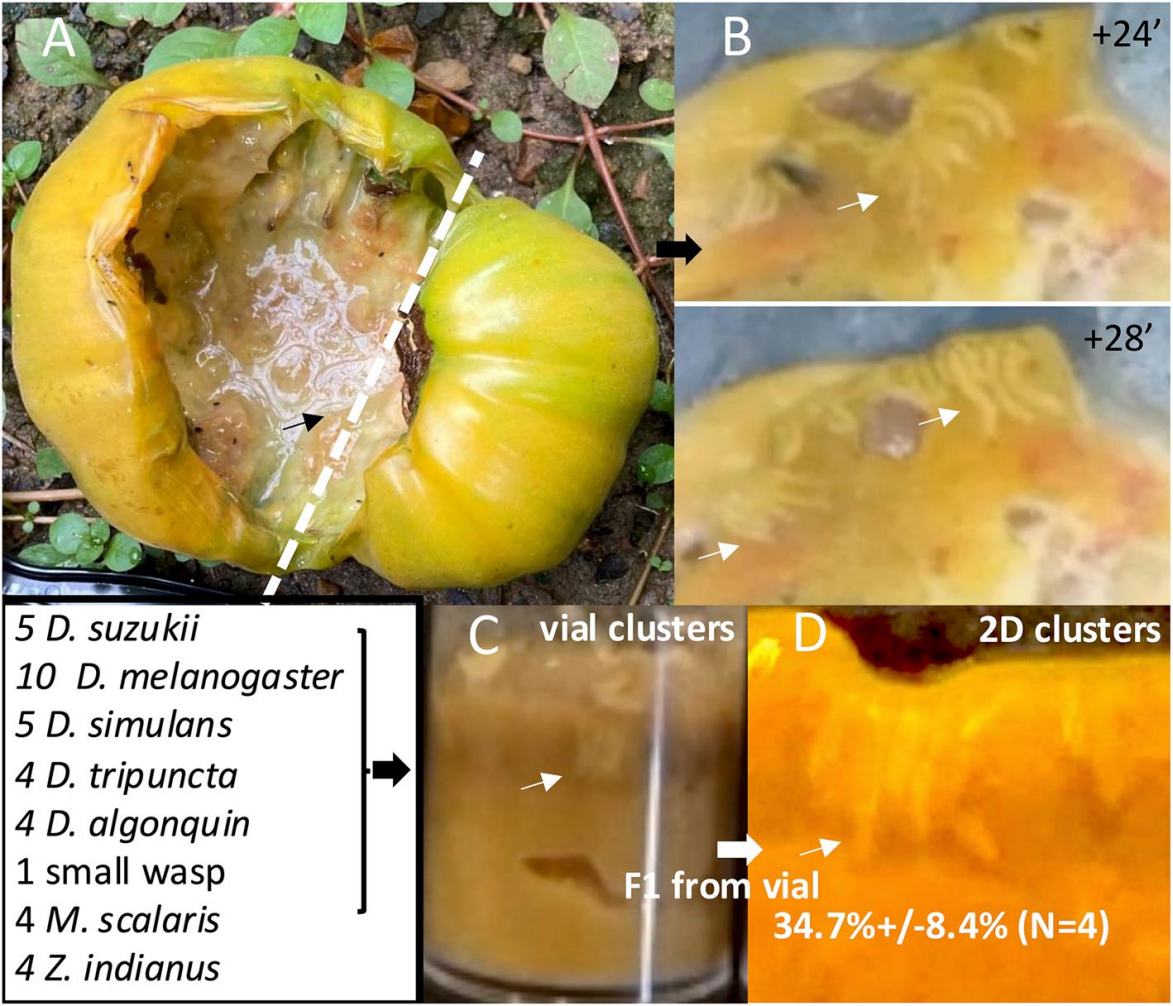
**a** A tomato that had fallen from a vine had ruptured and the center liquified with visible small larvae. The tomato was cut (dashed white line) and a 50 × 75 mm glass slide was placed against the liquified center. Cluster-like larval groups were seen almost immediately. These were video recorded.** b** Two still photos from a video shows what fulfill the definition of a cluster (Dombrovski et al. 2017). A 2 cm cube of material including the cluster in B was removed and placed in a vial.** c** Clusters from the tomato sample were seen in the vial. These larvae were incubated to adults and 7 species of flies and one small wasp hatched. These were allowed to reproduce and lay eggs in a separate vial. From this F1 vial, only 5 species hatched.** d** Samples from this mixed species F1 vial were tested for clustering in 2D assays using CS processed food. Adults hatching from this vial were separated by species into breeding pairs

A tomato in one of the authors’ backyards fell from a vine and split open in the summer of 2021 in central Virginia. Within a day it was swarmed with various small flies and wasps. Over the next two days, the center became liquid, and many small larvae could be seen. The tomato was sectioned in half, and a glass slide was placed against the liquid center and video recorded (Fig. 1a, c). Clusters were seen to form and lasted about 12 h (Fig. 1b). The larvae in the cluster (Fig. 1b) were scooped out and placed in a processed Caltech-food fly vial in which clusters continued to form (Fig. 1c). Over the next two weeks pupae formed and hatched into adults. These were identified morphologically as 7 species of fly (Miller et al. 2017) and were placed in a new vial. Grouped larvae from this vial were tested in 2D assays and formed good clusters (Fig. 1d, Fig. s1b). These were all placed back in the vial and grown to adults. From this, breeding pairs of all species were established from which one line from each of 5 species was established. To establish inbred lines, single male–female sib pairs were crossed for 10 generations. Two species, *D. tripuncta* and *D. algonquin*, were lost after the first passage. 4 of these species are closely related drosophilids which consume rotting fruit and one, *Megaselia scalaris*, or Scuttle fly, is a generalist in diet.

**Fig. 2:**
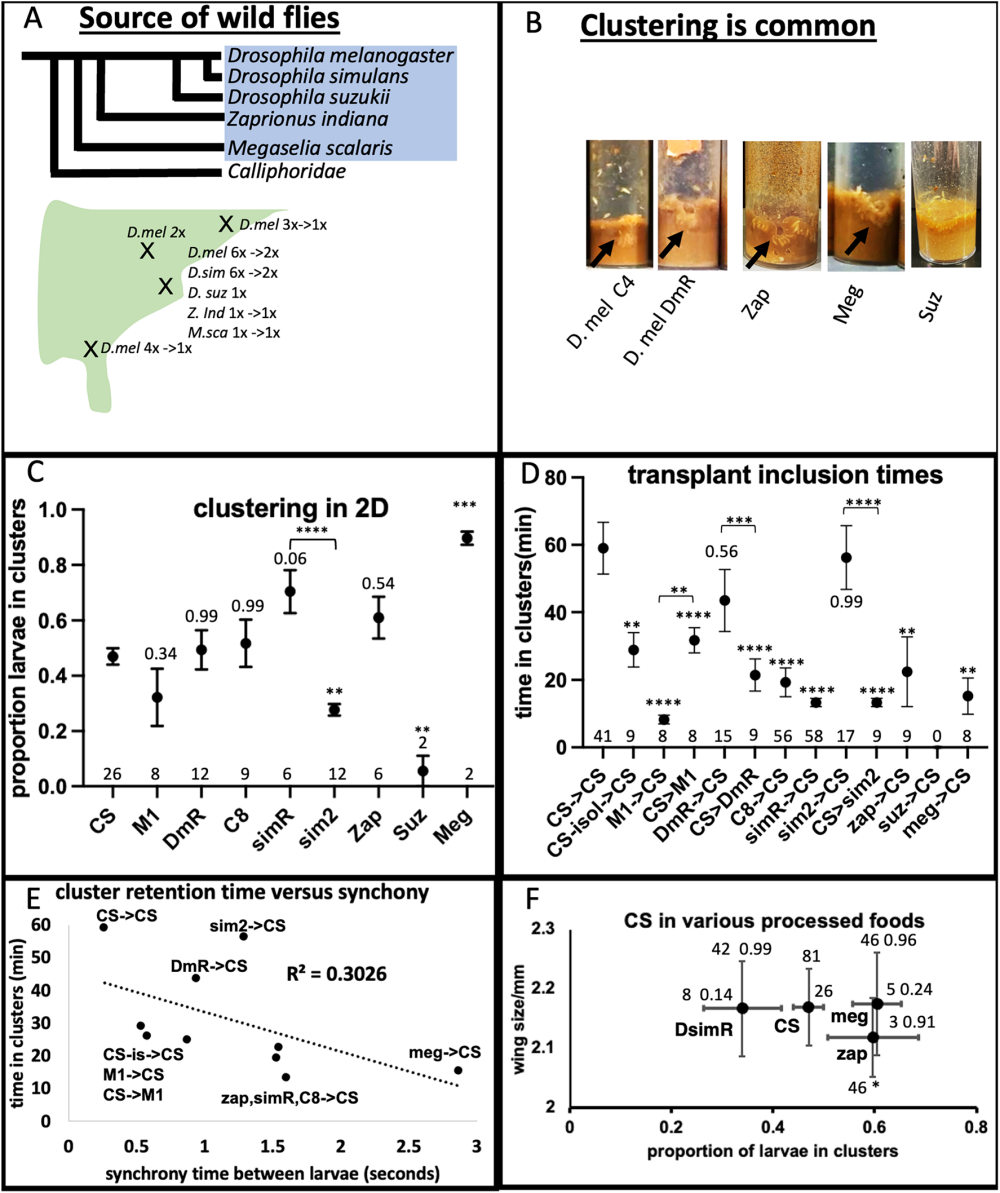
**a** Each species was grouped in separate vials and produced 5 lines of 5 species. From each line, single sibs were chosen and crossed. This was done > 10 times to make inbred lines. These were combined with 19 previously established inbred lines (see materials) for further study. All together, these wild flies originate from 4 locations in the Eastern US. The relationships of the 5 species are indicated and the ones used in this study are shaded in blue. *Calliphorids*, which might also cluster (Scanvion et al. 2018), were not used in this study. **b** At least 50 adults from each of the 24 lines were placed in egg cups and 24 h old larvae were obtained. About 200 of these larvae were placed in CS-processed vials and clustering was monitored over a few days. Clusters were observed in all vials except that of *D. suzukii*. See Fig. s1c. 9 lines of 5 species were chosen for further study. **c** Larvae were allowed to cluster in CS-processed vials as described (Dombrovski et al. 2017). 40 larvae were removed and placed in a 2D apparatus with CS-processed food. The percentage of larvae in clusters at 4,5 and 6 h was averaged. All larvae cluster with the exception of *D. suzukii*. Almost all larvae of *Megaselia* larvae are in clusters. Points indicate average values and error bars represent the standard error. The number of 2D samples used is indicated on the X-axis. Statistical probabilities were calculated by ANOVA, after normality tests, followed by Tukey’s method. The probabilities compared to CS are indicated with significance marked by P < 0.01 ** and P < 0.001 ***. A T-test was performed between simR and sim2 and is significant P < 0.0001. **d** Clustering residing times of transplanted larvae. Third instar larvae were removed from crowded vials with clusters and labeled with food coloring. Single larvae were placed over clusters of host larvae and the residence time was measured as described (Dombrovski et al. 2017, 2019). With the exception of M1 and suz, all spend 20-60’ in clusters. suz larvae do not enter CS clusters but data is included on the graph for clarity. There is also asymmetry in many transplantations, like M1- > CS/CS- > M1, DmR- > CS/CS- > DmR and sim2- > CS/CS- > sim2. Points indicate average values and error bars represent the standard error. The number of 2D samples used is indicated on the X-axis. Statistical probabilities were calculated by ANOVA, after normality test, followed by Tukey’s method. The probabilities compared to CS- > CS are indicated with significance marked by P < 0.01 **, P < 0.001 *** and P < 0.0001 ****. A T-test was performed between the 3 reciprocal pairs. **e** Inter-larval synchrony time was measured for transplanted larvae and compared to residing time in clusters. When measured for the different wild types, synchrony explains some of the variance in cluster residence time. However, some cases of long residence time, like sim2- > CS, seems to happen without a high degree of synchrony. **f** To test for the potential effects of species-specific food processing, food from 3 species was used to host CS 2D clusters. Plotted is the proportion of CS larvae clustering and resultant wing size of emerging adults from matched vials. Compared to food processed by CS, that processed by *Megaselia*, *Zaprionus* and *simulans* supports CS is clustering and gives healthy adult wing sizes. Only *Zaprionus* food gives lower wing sizes for CS. The data points are the averages of the indicated number of samples. The error bars indicate standard deviation for wing size and standard error for clustering. Statistics were calculated by normality tests followed by ANOVA and Tukey’s test. The probabilities are indicated and * is P < 0.05

Because of the potential that some members of the wild cluster could be sibs, other wild caught lines were obtained to broaden the repertoire (Fig. 2a, Fig. s1c, see methods). A number of extra *D.melanogaster* and one *D. simulans* lines, captured in different locations of the Eastern United States and established in by different labs were included in these studies. This ensures that the behaviors observed are not restricted to the initial collection location. Of 25 wild caught lines of 5 species, all cluster in vials except for *D. suzukii* (Fig. 2b, Fig. s2a). To quantify clustering further, 40 larvae were removed from vial clusters and placed in 2D assays using *D.melanogaster* CS pre-processed food as described (Dombrovski et al. 2017) (Fig. 2c). With the exception of *D.suzukii*, all cluster and some, like *Megaselia*, are almost 100% in clusters (Fig. s2b). Some lines, like the two *D. simulans* lines, vary significantly from each other arguing against a simple species pattern.

To test for the idea of co-clustering, transplantation experiments were conducted of larvae from species shown in (Fig. 2) into *D.melanogaster* CS clusters. In these experiments, food-coloring tagged larvae were placed into pre-established CS clusters as described (Dombrovski et al. 2017). CS host clusters were established from clusters in vials and on CS-preprocessed food. For intruders, larvae were removed from clusters in vials, placed in food coloring for 30’ and placed over CS clusters. The residing time was measured as how many minutes an intruder spent in a host CS cluster (Fig. s2c, d, e, f). Times vary greatly and do not necessarily correlate with single species clustering measures. For instance, sim2 does not cluster well with itself but resides in CS clusters as well as CS larvae themselves (Fig. 2c, d). High resolution movies were made of transplants and the inter-larval delay time in movement was used to measure the degree of synchronization (Dombrovski et al. 2017) (Fig. s2f, g). Plotting the synchrony time versus residence time in clusters reveals a relationship (Fig. 2e). The shorter the synchrony time, which means more alignment of the transplanted larva to the hosts, the longer the intruder resides.

For all measures of these wild caught flies, CS processed food was used. The clustering parameters for this food have been well established (Liao et al. 2024). To examine the potential role of species-specific processing, food was processed as described (Dombrovski et al. 2017) but using simR, zap or meg as the feeders. CS 2D clustering was examined on all three food types and compared to that processed by CS. In addition, 40 L2 CS larvae were loaded into a vial with food processed by one of the 3 wild types, and wing size from emerging adults were measured (Fig. 2f). None of the clustering measures were significantly different from that on CS food (Fig. 2f), although simR is trending to lower clustering. For wing size, zap food produces slightly lower wing size but not simR or meg. This means that while food processing can have an effect on clustering of other species, the effect is small.

**Fig. 3:**
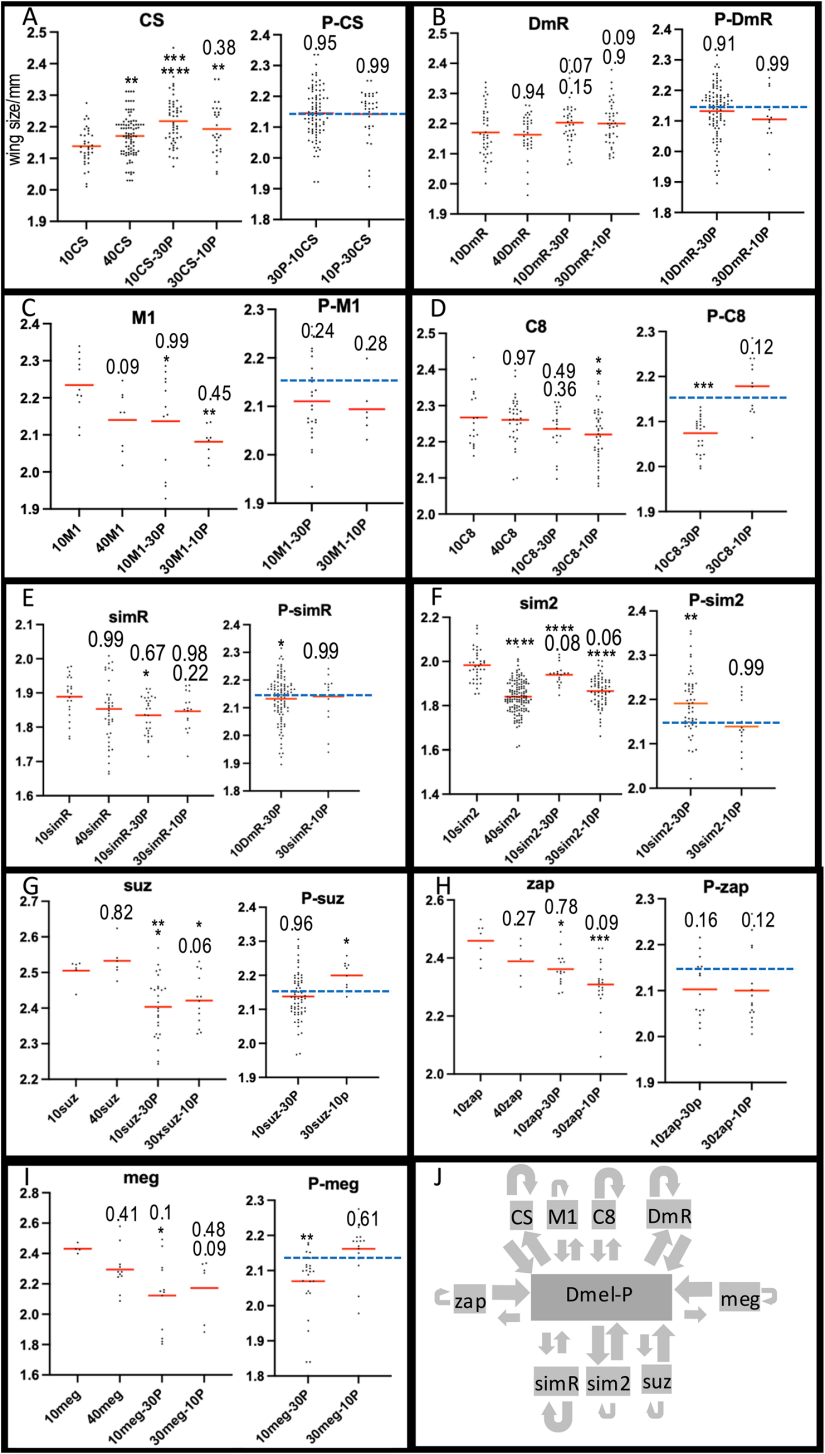
Larva fitness was measured by female adult wing size (Y axis). Second instar larvae were placed in a vial, as 10x, 40x, 10 × and 30 × wild type *D.melanogaster* host ‘P, and 30 × with 10xP. The effects of intra-specific clustering can be seen in any change from 10 × to 40x. The effects of heterospecific blends can be measured by comparing 40 × with either 10–30 or 30–10 blends with P. The effects on P are summarized in the right graph for each panel. The blue line represents the average 40xP wing size. The individual wing sizes are shown with the red line marking the mean. Statistical significance was measured by ANOVA post a normality test followed by Tukey’s method. The upper of the two probabilities compares to 40 × and the lower to 10 × of the same kind of larvae. P < 0.05 *, P < 0.01 **, P < 0.001 *** and P < 0.0001 ****. For P host measurements, the probability is compared to 40xP (Liao et al. 2024). **a** CS: data is replotted and analyzed from before (Liao et al. 2024). CS gains more from P than P from CS. **b** DmR neither gains nor loses to P. **c** M1 loses fitness to P and P remains the same. **d** C8 and P lose fitness in blends. **e** simR and P lose fitness in some blends. **f** sim2 loses in self clustering, and also loses in blends while P gains. **g** suz loses in blends while P gains. **h** zap loses in blends while P remains neutral. **i** meg and P lose fitness in some blends. **j** Summary of fitness gains or losses in blends with P for each of the 9 lines tested. An arrow pointing to the P host indicates a gain in fitness of P and an arrow to the test line a gain of that line. The self referential arrows indicate gains of self clusters. The large arrows indicate gains while the small arrows either a loss or neutral effect. There is not a species pattern to the gains or losses

To examine the effects of larval mixed foraging on adult fitness, female wing size was used, as described (Dombrovski et al. 2020). Wing size is a good indicator of body size which relates to fecundity (Gilchrist and Partridge 1999). For each line, 10 and 40 L2 larvae were loaded into separate vials based on the finding that wing size is larger at 40 compared to 10 animals per vial (Liao et al. 2024). An increase in wing size going from 10 to 40 indicates an advantage with greater numbers (Liao et al. 2024).Larvae were then mixed with the benchmark lab ‘P’ strain which clusters well with CS (Liao et al. 2024). This strain is a lab-based transgenic and inbred line which has been used as an alternative to CS (Liao et al. 2024). These were mixed at 10:30 and 30:10 ratios such that there were 40 larvae per vial. Wing size of the test line and P was established for each experiment (Fig. 3). This allows an estimation of the value of clustering, the value of mixing with the lab P strain and the effects on P. For comparison, data from (Liao et al. 2024) are replotted in this format in Fig. 3a. CS larvae produced bigger wings when in groups of 40, either alone or mixed with P and P is equally advantaged by this arrangement (Fig. 3a). This is schematized in Fig. 3j where arrow size indicates gain (large arrow) or neutral/loss (small arrow). For the 3 *D.melanogaster* lines, M1, DmR and C8, each behaves in a different manner (3a, b, c, d). In general, CS and DmR show mutualistic gain with P while M1 and C8 do not.

Similarly for *D.simulans*, one line shows a mutualistic gain with P, (sim2, Fig. 3f) while the other does not (simR, Fig. 3e). All three of the other species, *M. scalaris*, *D.suzukii* and *Z. indianus* show mixed interactions with P. P gains from all three in some of the mixes.

**Fig. 4:**
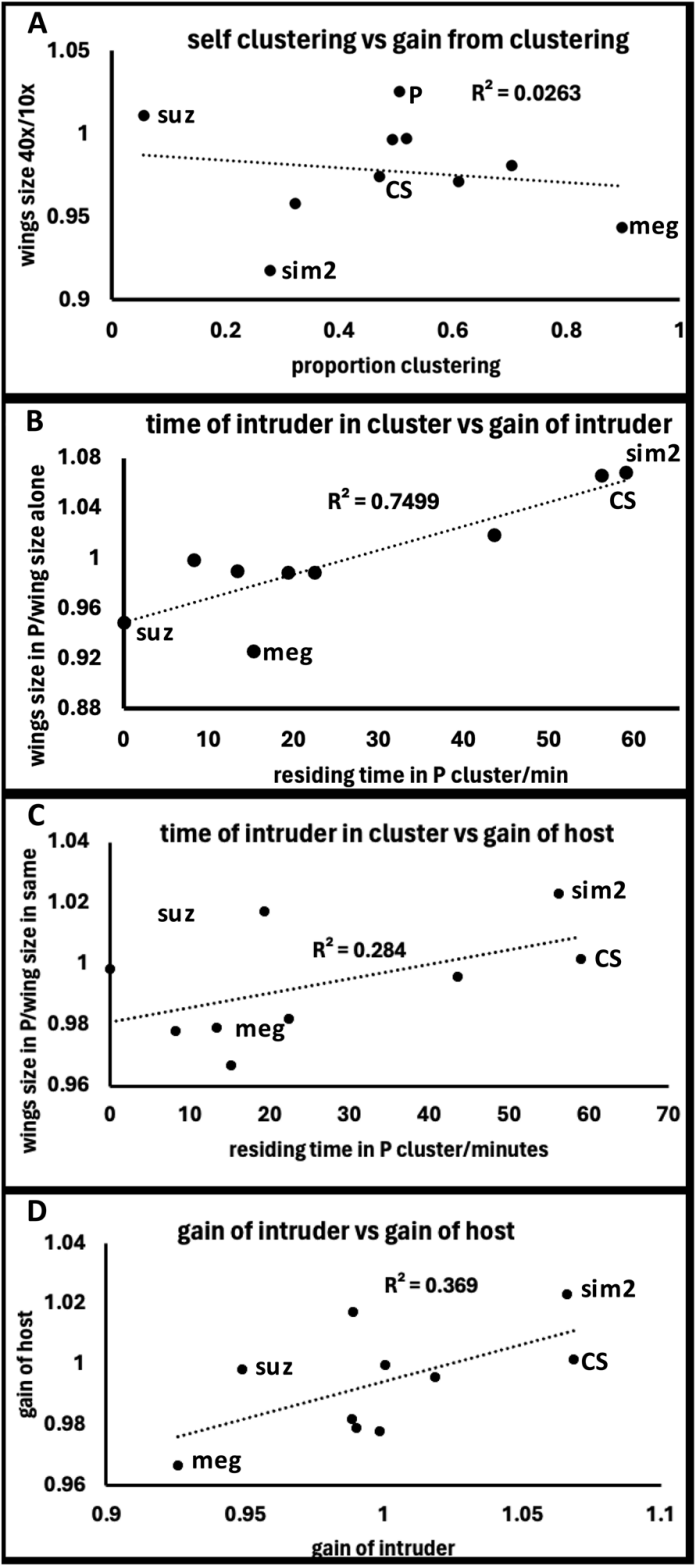
Clustering and fitness data from wild caught flies were assembled and correlations examined. Four selected lines are indicated for each graph. **a** The relationship between the amount of clustering any line conducted with itself was compared to the gain from that behavior. The gain from mono-species clustering was measured by wing size for 40 × larvae in a vial divided by that from only 10 larvae. This was compared to proportion of larvae that cluster in 2D. **b** The gain in fitness of an intruder in P was measured by wing size at 10/30 blend compared to 40 alone. This is plotted against time spent by an intruder in a 2D P cluster. **c** The same relationship in 4B is now plotted but now as the gain of the host versus the time spent by the intruder in that host cluster. **d** Gain of intruder is plotted against the gain of the host

To examine for broader relationships, wing size and clustering data from. s 2 and 3 were compared. To examine the role of self-clustering, the wing effects of clustering (wing size at 40 compared to wing size at 10, first two columns in Fig. 3) were compared to how much clustering each line does (Fig. 2c). No relationship is seen (Fig. 4a). The relationship between time spent by an intruder in a cluster (Fig. 2d) and gain from that cluster (wing size 10/30P divided by 40, or third column in Fig. 3 divided by second) gives a strong relationship. CS and sim2 spend most time in P and gain the most while suz spends least time and gains the least. Interestingly, plotting the same x axis against the gain for the host P (Fig. 4c, P wings in 10/30P divided by 40xP alone) also shows a relationship. If the intruder gains, so does the host. This is further clarified in Fig. 4d by plotting the gain of the intruder over the gain of the host. If the intruder gains, so does the host.

## Discussion

This study provides evidence that fly larvae likely cluster in the wild as a one such cluster was documented, and when reconstituted in the lab, continues to demonstrate this behavior. Larval clusters are therefore not likely to be lab artifacts from long-term culture in vials. In addition, like well characterized *Caliphora* larval groups on a carcass (Scanvion et al. 2018; Charabidze et al. 2021), rotting fruit larval clusters can be mixed species. However, this study also indicates that there is high genetic variance in how individual lines cluster with each other that is not explained by cross species features. Clustering in the wild is therefore likely to be a complex mixture of different behaviors that still succeeds in collective foraging. Additionally, clustering can provide mutual benefits when two different lines are mixed.

There are many caveats to this study. Only a small number of lines have been used and the parameters of clustering derived from *D.melanogaster* have been assumed to apply to the other species (Liao et al. 2024). There are likely to be differences in life cycle, clustering parameters, food processing, growth rates and microbiomes between species. However, the high variance between inbred lines of two species suggests a strong genetic component.

The fact that many of these lines were obtained from the same cluster on the same fruit likely selected for co-clustering.

Of the 24 lines examined in this study, only *D. suzukii* does not cluster. There is a possible mismatch in food as *D.suzukii* prefers fresh fruit and not the more decomposed media used in this study. One of the two isolated *D.simulans* lines, one, sim2, does not cluster well with itself. However, a symbiotic relationship has been proposed where *D.suzukii* can pioneer fresh fruit digestion followed closely by *D.melanogaster* general processing (Solomon et al. 2019). Speculatively, the split tomato from which many of these lines were obtained, may have been compromised on the vine by *D.suzukii* and followed by colonization from the other species. Given the extensive crop damage that invasive *D.suzukii* does (Tungadi et al. 2023), it will be very important to explore the details of the advantages and disadvantages of clustering to this pest along with other species.

Clustering is thought to provide advantages to larvae through mixed social exodigestion (Gregg et al. 1990; Dombrovski et al. 2017, 2020) and likely microbiomic farming and food mixing. This should, in general be advantageous to all members unless there is a mismatch in the nature of food processing or ability to synchronize as a group. The ability to remain a part of a group is directly related to fitness outcomes (Dombrovski et al. 2020; Williamson et al. 2021). The nature of food processing seems to be relatively similar across species, as CS larvae cluster on food processed by 3 other species (Fig. 2f). Among three of the selected groups used in this study, there is no substantial alteration of the processed food by one species such that only they can cluster and/or survive. The evidence in this study points to mixed mutual cooperative foraging. In general, clustering as a mixed species can be as beneficial as a pure species. The more gain seen in an intruder (CS, sim2) the more gain to the host and the less gain (suz, meg) the less the gain to the host (Fig. 4d). The degree of co-clustering is directly related to the gain of the intruder (Fig. 4b). What is less clear is how some of these larvae succeed in co-clustering. Previous studies have indicated that co-clustering requires learning (Dombrovski et al. 2017, 2019) and matched environmental conditions (Williamson et al. 2021). However, obligate cheater larvae with attenuated salivary glands integrate better than host-cluster members and this might be due to other factors such as perception of nutritional state (Liao et al. 2024). Depending on the conditions, the blend of larvae on a substrate will be initiated by ovipositional choices from adult females (Zhang et al. 2020). Different fruits can establish different microbiomes (Foster and Fogleman 1993) and this could prime the ensemble of larvae through directed oviposition (Becher et al. 2012). A reasonable question is why larvae are so broad on accepting of other members of different species seen in this study and in carcass digestion (Scanvion et al. 2018). What can be concluded from this study is that wild-collected lines are capable of clustering, and have mixed degrees of clustering in the lab, with or without non-conspecifics. Although the exact environmental factors that bring about clustering in a natural setting are unknown, it is likely in part due to pre-digestion of the food, the blend of individuals and how they signal in a group. More focused studies on a subset of these lines will likely shed further light in how cooperative foraging occurs.

## Supplementary Information

Below is the link to the electronic supplementary material.

Supplementary file1 Schematic diagram of workflow for obtaining the wild caught lines (TIFF 60271 KB)

Supplementary file2 Video of an outside cluster on a tomato. This was collected and the resultant larvae used to make lines for this study (MP4 26382 KB)

Supplementary file3 Video of 2D clustering of F1 mixed larvae obtained from the wild tomato cluster (MP4 16662 KB)

Supplementary file4 Images of clusters in vials of 25 wild-caught fly lines of which 24 cluster (TIFF 289465 KB)

Supplementary file5 Example video of Megaselia scalaris clustering in 2D (MP4 152753 KB)

Supplementary file6 (TIFF 60255 KB)

Supplementary file7 Example video of transplanted Zap into CS 2D clusters (MP4 129037 KB)

Supplementary file8 Example video of transplanted sim2 in CS 2D clusters (MP4 120805 KB)

Supplementary file9 Example video of transplanted med in CS 2D clusters (MP4 132853 KB)

Supplementary file10 Example video of transplanted sim2 in CS 2D clusters at high resolution (MP4 386896 KB)

Supplementary file11 Example video of transplanted DmR in CS 2D clusters at high resolution (MP4 874845 KB)

## Data Availability

No datasets were generated or analysed during the current study.
