# Supplementary figures and images for: Do wild-caught fly larvae cooperatively forage?
Source: J Comp Physiol A Neuroethol Sens Neural Behav Physiol. 2024 Nov 26;211(2):199–208. doi: 10.1007/s00359-024-01724-3 (PMC12003498; doi:10.1007/s00359-024-01724-3)

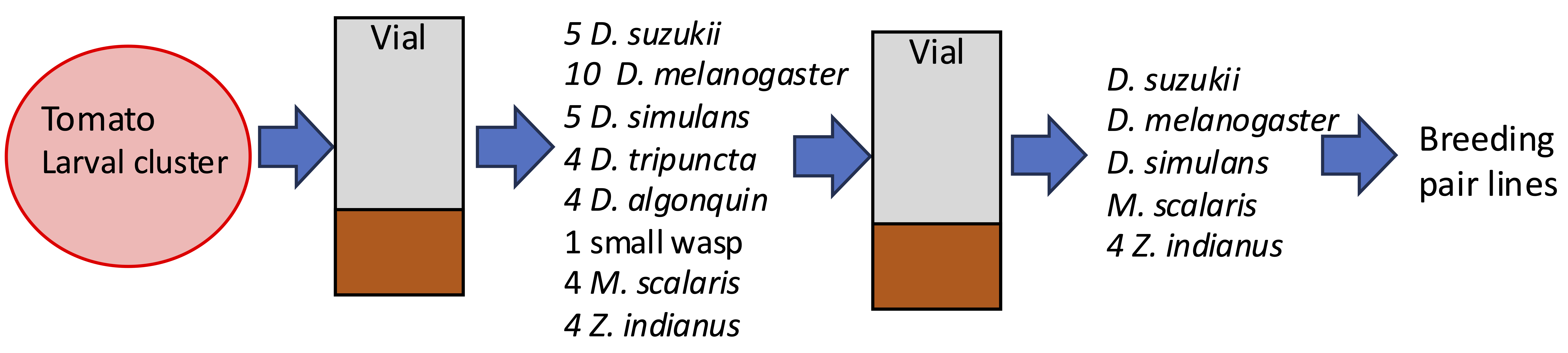

Supplement: Supplementary file 1 — Supplementary file1 Schematic diagram of workflow for obtaining the wild caught lines (TIFF 60271 KB) [file 359_2024_1724_MOESM1_ESM.tiff]

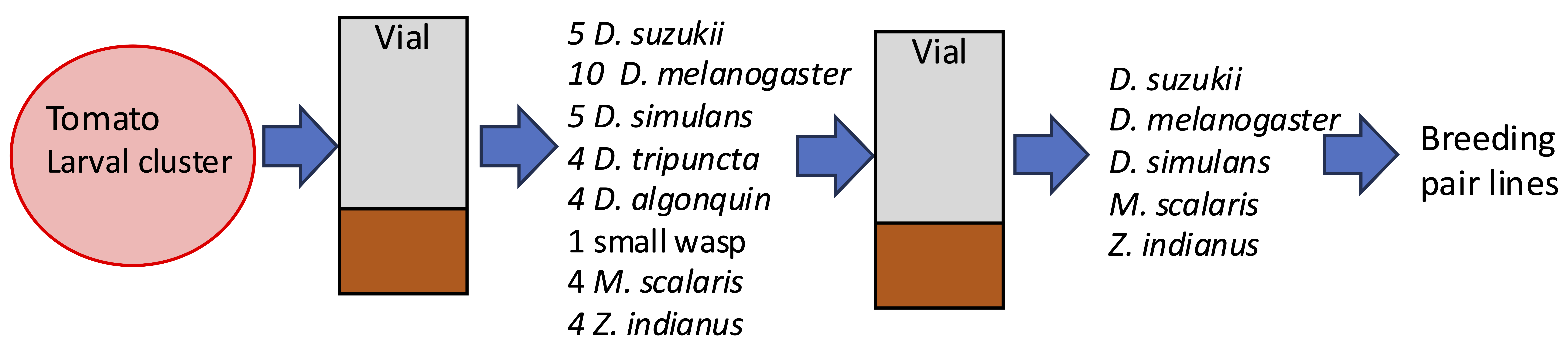

Supplement: Supplementary file 4 — Supplementary file4 Images of clusters in vials of 25 wild-caught fly lines of which 24 cluster (TIFF 289465 KB) [file 359_2024_1724_MOESM4_ESM.tiff]
